# Falls in Scottish homicide: lessons for homicide reduction in mental health patients

**DOI:** 10.1192/pb.bp.116.054924

**Published:** 2017-08

**Authors:** John H. M. Crichton

**Affiliations:** 1 School of Law, University of Edinburgh, Edinburgh, UK

## Abstract

The sustained fall in Scottish homicide rates follows crime reduction measures informed by the epidemiology of suicide. The violence reduction unit targeted young men carrying knives in public. The restriction of weapons immediately to hand appears to have caused an absolute fall in homicide just as suicide reduction was observed following changes to domestic gas supply. Further homicide reduction may be accomplished in the domestic setting with targeted changes in kitchen knife design in home safety planning for high-risk households. Most commonly homicides involving those in recent contact with mental health services in the UK have domestic characteristics and similar safety planning may be targeted at those with mental disorder and a history of violence.

The sustained fall in Scottish homicide rates to 10.65 per million in 2015–2016 marks a 60% fall in the homicide rate since the peak of 26.3 per million in 2005.^1,2^ The greatest fall in homicide involves encounters between young men in public places. The Violence Reduction Unit, formed in 2005, utilised a public health approach to target young men with educational programmes and stiff penalties aimed at achieving reduction in knife carrying outside the home.^3^ From 2005 to 2016 police in Scotland recorded a 69% fall in cases of offensive weapon carrying.^4^ From 2008 to 2015, Scottish hospitals saw a 63% fall in admissions and a 50% reduction in deaths arising from assaults with a sharp object.^5^ Limiting the availability of a lethal weapon immediately to hand outside the home has been associated with a dramatic decline in homicide and serious injury.

The targeting of knife carrying in Scotland is an example of situational crime reduction, a highly successful approach inspired by the fall in suicide following changes to domestic gas supply.^6^ The model, aimed at increasing the difficulty of accomplishing a criminal act, arose from observing the marked decline in the UK suicide rate associated with the change from coal gas to natural gas.^7^ A major means of suicide – placing one's head in an unlit oven and breathing in the gas – abruptly disappeared. Not only was there a dramatic fall in carbon monoxide suicides but a fall in the number of suicides overall. It can be inferred then that there are a group of individuals who, while apparently committed to dying by suicide, can be deviated from a life-ending course of action by a seemingly trivial inconvenience.

Despite the fall in overall Scottish homicides, this has not been observed in homicides associated with those in recent contact with mental health services.^8^ The stereotype of homicides associated with mental disorder involving stranger victims and unusual weapons in public places is false. In a 10-year review of 870 UK homicides carried out by current or recent users of mental health services, the homicide victim was a spouse or ex-spouse in 21% of cases, another family member in 18% and other acquaintances 46%; 15% were stranger victims, as opposed to 24% stranger victims for all homicides.^8^ Similar findings were observed in a 15-year series of 271 homicides carried out by mental health patients in England, where 42% occurred in the shared home of the victim and perpetrator, 25% at the victim's home and 4% in the perpetrator's home.^9^ To test the hypothesis that ordinary objects were used in such homicides, this cohort was re-examined: 45% overall involved a knife and of those knives that could be identified 85% were kitchen knives.^10^ Homicides associated with mental disorder typically have domestic characteristics, involving family or acquaintances in a domestic setting, not the homicide type targeted in the Scottish campaign.

The evidence would suggest that limiting weapon carrying in public reduces homicides outside the domestic setting, but how can this be achieved within the home where kitchen knives are required? Long sharp-tipped knives have limited specific culinary utility and alternative designs are available.^11^ One design has an ‘r’ shaped tip and has been demonstrated as being as effective as wearing an anti-stab vest in a thrust to the torso (details available from the author on request), although similar benefits to safety may be achieved with a rounded or square tips.

Perhaps murder mythology in fiction and in the news media, with its emphasis on the exceptional and planned murder, obscures the possible benefits to changes to knife design, which may reduce the injury of unplanned acts of impulsive violence within the home.^12^ Yet there is media bias for reporting the unusual, stranger homicide involving those with mental disorder.^13^ This obscures the role of promoting home safety measures utilised in domestic violence reduction for those with mental disorder and a history of violence and weapon use. Such a public health approach could be criticised for restricting freedom and would not stop a planned act of violence. Conversely, this may provide a way of generalising the benefits observed in Scotland to a domestic situation. The role of simple barriers to immediate weapon use in homicide reduction may also indicate strategies for violence reduction in other contexts.
